# Evaluating the risk of patient re-identification from adverse drug event reports

**DOI:** 10.1186/1472-6947-13-114

**Published:** 2013-10-05

**Authors:** Khaled El Emam, Fida K Dankar, Angelica Neisa, Elizabeth Jonker

**Affiliations:** 1 Children’s Hospital of Eastern Ontario Research Institute, 401 Smyth Road, Ottawa, Ontario, K1J 8 L1, Canada; 2 Pediatrics, Faculty of Medicine, University of Ottawa, Ottawa, Ontario, Canada

## Abstract

**Background:**

Our objective was to develop a model for measuring re-identification risk that more closely mimics the behaviour of an adversary by accounting for repeated attempts at matching and verification of matches, and apply it to evaluate the risk of re-identification for Canada’s post-marketing adverse drug event database (ADE).Re-identification is only demonstrably plausible for deaths in ADE. A matching experiment between ADE records and virtual obituaries constructed from Statistics Canada vital statistics was simulated. A new re-identification risk is considered, it assumes that after gathering all the potential matches for a patient record (all records in the obituaries that are potential matches for an ADE record), an adversary tries to verify these potential matches. Two adversary scenarios were considered: (a) a mildly motivated adversary who will stop after one verification attempt, and (b) a highly motivated adversary who will attempt to verify all the potential matches and is only limited by practical or financial considerations.

**Methods:**

The mean percentage of records in ADE that had a high probability of being re-identified was computed.

**Results:**

Under scenario (a), the risk of re-identification from disclosing the province, age at death, gender, and exact date of the report is quite high, but the removal of province brings down the risk significantly. By only generalizing the date of reporting to month and year and including all other variables, the risk is always low. All ADE records have a high risk of re-identification under scenario (b), but the plausibility of that scenario is limited because of the financial and practical deterrent even for highly motivated adversaries.

**Conclusions:**

It is possible to disclose Canada’s adverse drug event database while ensuring that plausible re-identification risks are acceptably low. Our new re-identification risk model is suitable for such risk assessments.

## Background

There is increasing pressure to make raw health data on individuals more generally available for research, policy, and commercial purposes [1]. There are also pressures on governments to disclose more data through Access to Information requests [2]. However, without obtaining the individuals’ consent a priori for such disclosures, such data needs to be appropriately de-identified^a^.

Recent incidents have demonstrated that publicly available, and ostensibly de-identified data, can still allow the re-identification of individuals [3]. In one notable example, Health Canada’s Adverse Drug Event (ADE) database (historically referred to as CADRIS, but has recently been renamed [4,5]) was obtained through an Access to Information request by a national broadcaster. The national broadcaster was then able to re-identify a 26 year old female patient who died while taking a particular medication by linking her ADE record with the publicly available obituaries, contacted her family, and broadcast a story about the adverse effects of that drug referring to the girl’s death as an example [6]. The publicity coincided with Health Canada issuing a safety advisory about the medication in question [7].

Drug and device manufacturers are required to report adverse reactions to regulators. Otherwise, reporting is voluntary and comes from physicians and patients. In the US the equivalent system is AERS (Adverse Event Reporting System) [8]. Regulators in Canada and the US make ADE data publicly available through Access to Information/Freedom of Information requests [5,9], and these data have been used by researchers [9,10]. However, as the Canadian example above illustrates, the re-identification of patients whose death has been reported in an ADE database is plausible, and privacy concerns around the disclosure of these reports have resulted in lengthy and costly litigation in Canadian federal court [6].

Given the utility of ADE databases for researchers and the media, it is important to make the data publicly available, but in a manner that ensures individual patients cannot be re-identified. Since measures existing at the time did not prevent the re-identification of an ADE record by a national broadcaster, it is important to analyze this attack and prevent similar future re-identification attempts.

In this paper we present a new more general re-identification risk model that extends previous models by covering the actual behavior of the broadcaster. The new model assumes some degree of effort from the adversary in validating potential matches. The model will be presented in detail in the next section. We then performed a simulation to evaluate the risk of re-identification of Canadians from public ADE reports. We focus on reports where the outcome was death because there is evidence of successful re-identification attacks where the outcome is death.

### Definitions

We start off by providing some definitions that are used in our model development.

#### Categories of variables

It is useful to differentiate among the different types of variables in a disclosed data set. The way the variables are handled when evaluating re-identification risk will depend on how they are categorized. We make a distinction among four types of variables [11,12], and these are illustrated in the hypothetical claims data in Table 1:

**Table 1:** Example data: this hypothetical example table is used to illustrate a number of concepts that we use throughout our analysis

	**Identifying variable**		**Quasi-identifiers**		**Sensitive variables**		**Other variables**
**ID**	**Name**	**Telephone number**	**Sex**	**Year of birth**	**Lab test**	**Lab result**	**Paydelay**
1	John Smith	(412) 688-5468	Male	1959	Albumin, Serum	4.8	37
2	Alan Smith	(413) 822-5074	Male	1969	Creatine kinase	86	36
3	Alice Brown	(416) 886-5314	Female	1955	Alkaline Phosphatase	66	52
4	Hercules Green	(613) 763-5254	Male	1959	Bilirubin	Negative	36
5	Alicia Freds	(613) 586-6222	Female	1942	BUN/Creatinine Ratio	17	82
6	Gill Stringer	(954) 699-5423	Female	1975	Calcium, Serum	9.2	34
7	Marie Kirkpatrick	(416) 786-6212	Female	1966	Free Thyroxine Index	2.7	23
8	Leslie Hall	(905) 668-6581	Female	1987	Globulin, Total	3.5	9
9	Douglas Henry	(416) 423-5965	Male	1959	B-type natriuretic peptide	134.1	38
10	Fred Thompson	(416) 421-7719	Male	1967	Creatine kinase	80	21
11	Joe Doe	(705) 727-7808	Male	1968	Alanine aminotransferase	24	33
12	Lillian Barley	(416) 695-4669	Female	1955	Cancer antigen 125	86	28
13	Deitmar Plank	(416) 603-5526	Male	1967	Creatine kinase	327	37
14	Anderson Hoyt	(905) 388-2851	Male	1967	Creatine kinase	82	16
15	Alexandra Knight	(416) 539-4200	Female	1966	Creatinine	0.78	44
16	Helene Arnold	(519) 631-0587	Female	1955	Triglycerides	147	59
17	Almond Zipf	(519) 515-8500	Male	1967	Creatine kinase	73	20
18	Britney Goldman	(613) 737-7870	Female	1956	Monocytes	12	34
19	Lisa Marie	(902) 473-2383	Female	1956	HDL Cholesterol	68	141
20	William Cooper	(905) 763-6852	Male	1978	Neutrophils	83	21
21	Kathy Last	(705) 424-1266	Female	1966	Prothrombin Time	16.9	23
22	Deitmar Plank	(519) 831-2330	Male	1967	Creatine kinase	68	16
23	Anderson Hoyt	(705) 652-6215	Male	1971	White Blood Cell Count	13.0	151
24	Alexandra Knight	(416) 813-5873	Female	1954	Hemoglobin	14.8	34
25	Helene Arnold	(705) 663-1801	Female	1977	Lipase, Serum	37	27
26	Anderson Heft	(416) 813-6498	Male	1944	Cholesterol, Total	147	18
27	Almond Zipf	(617) 667-9540	Male	1965	Hematocrit	45.3	53

##### Directly identifying variables

One or more direct identifiers can be used to uniquely identify an individual, either by themselves or in combination with other readily available information. For example, there are more than 200 people named “John Smith” in Ontario (based on a search in the White Pages), therefore the name by itself would not be directly identifying, but in combination with the address it would be directly identifying information. A telephone number is not directly identifying by itself, but in combination with the readily available White Pages it becomes so. Other examples of directly identifying variables include email address, health insurance card number, credit card number, and social insurance number. These numbers are identifying because there exist public and/or private databases that an adversary can plausibly get access to where these numbers can lead directly, and uniquely, to an identity. For example, Table 1 shows the names and telephone numbers of individuals. In that case the name and number would be considered as identifying variables.

##### Indirectly identifying variables (quasi-identifiers)

The quasi-identifiers are the background knowledge variables about individuals in the disclosed data set that an adversary can use, individually or in combination, to probabilistically re-identify a record. If an adversary does not have background knowledge of a variable then it cannot be a quasi-identifier. The manner in which an adversary can obtain such background knowledge will determine which attacks on a data set are plausible. For example, the background knowledge may be available because the adversary knows a particular target individual in the disclosed data set, an individual in the data set has a visible characteristic that is also described in the data set, or the background knowledge exists in a public or semi-public registry.

Examples of quasi-identifiers include sex, date of birth or age, locations (such as postal codes, census geography, information about proximity to known or unique landmarks), language spoken at home, ethnic origin, aboriginal identity, total years of schooling, marital status, criminal history, total income, visible minority status, activity difficulties/reductions, profession, event dates (such as admission, discharge, procedure, death, specimen collection, visit/encounter), codes (such as diagnosis codes, procedure codes, and adverse event codes), country of birth, birth weight, and birth plurality.

For example, Table 1 shows the patient sex and year of birth (from which an age can be derived) as quasi-identifiers.

##### Sensitive variables

These are the variables that are not really useful for determining an individual’s identity but contain sensitive health information about the individuals. Examples of sensitive variables are laboratory test results and drug dosage information. In Table 1 the lab test that was ordered and the test results are the sensitive variables.

##### Other variables

Any variable in the data set which does not fall into one of the above categories falls into this ‘catch all’ category. For example, in Table 1 we see the variable PayDelay, which indicates how long (in days) it took the insurer to pay the provider. In general, this information is not considered sensitive and would be quite difficult for an adversary to use for re-identification attack purposes.

In the ADE database there were no direct identifiers, but it does have a number of quasi-identifiers as explained below. Our focus is on the re-identification risk from these quasi-identifiers.

#### Equivalence class

All the records that have the same values on the quasi-identifiers are called an *equivalence class*. For example, all the records in a dataset about 17 year old males are an equivalence class.

#### Identity vs attribute disclosure

There are two kinds of disclosure that are of general concern: identity disclosure and attribute disclosure [13,14]. The first is when an adversary can assign an identity to a record in the data set. For example, if the adversary would be able to determine that record number 3 belongs to patient Alice Brown using only the quasi-identifiers, then this is identity disclosure. The second type of disclosure is when an adversary learns a sensitive attribute about a patient in the database with a sufficiently high probability without knowing which specific record belongs to that patient [13,15]. For example, in Table 1 all males born in 1967 had a creatine kinease lab test. Assume that an adversary does not need to know which record belongs to Almond Zipf (record ID 17). Since Almond is male and was born in 1967 then the adversary will discover something new about him (that he had a test often given to individuals showing symptoms of a heart attack). This is attribute disclosure.

In analyzing the disclosure of the national ADE database, we only consider identity disclosure. There are a number of justifications for this.

Known re-identifications of personal information that have actually occurred are identity disclosures [3]. Furthermore, health privacy statutes and regulations in multiple jurisdictions, including the US Health Insurance Portability and Accountability Act (HIPAA) Privacy Rule and the Ontario Personal Health Information Act (PHIPA) only consider identity disclosure in their definitions of personal health information.

In the context of an Access to Information request, as is the case with Canadian ADE database, if the data custodian did more than was required by law in order to release less information, they can be taken to court by the data requestor. Therefore, it is even more critical to focus only on the requirements for managing identity disclosure risks only.

### Conceptual motivation

Measures of re-identification risk make assumptions about the method of attack that is used by the adversary. Most work on measuring the risk of re-identification assumes that the adversary will attempt to re-identify a single record in the disclosed database and then stop. That single attempt may succeed or fail. The adversary will use background information that she knows about someone in the disclosed database or that is obtained from public and semi-public registries.

In Canada, large public registries with full identity information (i.e. names) include obituaries, White Pages, Private Property Security Registration, and the Land Registry [16,17]. These public registries might also include other information such as the date of death, date of birth, geographic information about place of residence, and gender.

To illustrate, an adversary can select a record with unique values from an ostensibly de-identified ADE reports database which has been disclosed, then check for potential matches in public registries having full identity information by matching quasi-identifiers common to both databases. If there exists one potential match, then re-identification occurs [18,19]. On the other hand, if more than one potential match exists, and if the adversary is lacking any additional information, then re-identification with certainty cannot occur. Subsequently, the adversary might randomly select one of the potential matches as the final match. In this case, the adversary would not be certain of the correctness of the outcome, s/he would only have a probability of success associated with it. In the literature, although a random match (with probabilistic outcome) is not generally considered a re-identification, a low probability of success is still desired (0.2 is a common requirement [20-29]). The re-identification risk for an adversary who is content with a random match, referred to as the unmotivated adversary, depends on the size of the population equivalence class, and has been the focus of previous literature [11,30].

However, as illustrated in the broadcaster example, some adversaries are not content with uncertain matching. In the absence of unique matches (i.e., when faced with several potential matches), such motivated adversaries might take further measures to verify these matches in order to confirm the identity of the individual. For example, adversaries who are willing to assume some degree of effort to validate their potential matches could be journalists chasing a rewarding story, or firms marketing their medical products where the marketing process is costly per prospect. For each one of these examples, the degree of motivation can vary from highly motivated, to mildly motivated. In each case, the motivation is limited by practical and financial considerations. This scenario focuses on *certain* re-identification and is an extension of the uniqueness matching scenario.

The outcome of interest in our analysis is death. It is more difficult to re-identify records with other reported outcomes (e.g., nausea), and there is evidence that it can be, or has been, done for death outcomes. In addition to the Canadian broadcaster example of re-identification through obituaries, another study presents an example of how obituary data was used to re-identify uniques in de-identified pedigrees [18]. Therefore we only focus on deaths in the ADE database.

The re-identification of records belonging to patients who have died does not only affect the dignity of the deceased individual, but can also result in an invasion of privacy to their family and friends. For example, if a patient dies while taking a drug for a stigmatized infectious or hereditary disease then that may raise assumptions that the person’s family and/or friends may also have the same disease.

Consider Table 2 which shows ADE-like entries, and Table 3 which shows obituary entries. The quasi-identifiers that are used for matching are the patient’s age, gender, province, and date of death. In this example we use the ADE reporting date as a proxy for the date of death.

**Table 2:** Example of an extract from an adverse drug reaction database where the reported outcome was death, and a potentially matching extract from an obituary table

***Report ID***	***Age***	***Gender***	***Province***	***Report date***	***Drug name***	***Reaction***
1	42	F	British	5 May 1998	TALWIN FOR INJECTION	Suicide
			Columbia			
2	71	M	Alberta	2 Jan 1998	MAXERAN	Dehydration
3	34	M	Ontario	21 Sept 1998	Procainamide	Cardiac arrest
4	55	F	Quebec	1 Apr 1998	Rifampin	Congestive heart failure
5	38	F	Nova	25 Nov 2004	Tegretol	Non-accidental overdose
			Scotia			
6	44	M	Ontario	23 Oct 2006	Penicillin	Respiratory arrest
7	65	M	Quebec	24 Jun 2001	Morphine	Haemorrhage intracranial

**Table 3:** Example of an extract from an obituary table

**Name**	**Age**	**Gender**	**Province**	**Date of death**
John Smith	44	M	Ontario	23 Oct 2006
Alan Black	44	M	Ontario	23 Oct 2006
Hugh Tremblay	44	M	Ontario	23 Oct 2006
Joe White	44	M	Ontario	23 Oct 2006
Mary Lambert	65	F	Quebec	25 Nov 2004
Leslie Long	77	F	British Columbia	24 Jun 2001

We assume that the obituary has all deaths. The names in this table are fabricated names and do not knowingly match real individuals.

If an adversary selected the individual in report number 6 in Table 2, then there are four matches in the obituaries. Lacking any additional information, an unmotivated adversary would select one of these individuals at random and the probability of having a correct match is 0.25. It is important to note that in this case, the adversary cannot know if this match is correct or not, s/he only knows the probability of the match being correct.

In practice however, for a highly motivated adversary, such as the national broadcaster it would not be acceptable to have a breaking story about the wrong person. Knowing that there is a one in four chance that, say, Alan Black was the one on Penicillin and died from respiratory arrest would not be sufficient. Furthermore, the broadcaster would want to have a story around the specific family of the deceased individual. This makes it necessary to have certainty that the match is correct. Therefore, clearly such random matching would be insufficient and a method of verifying the potential matches to determine the correct one would be necessary.

In practice, the broadcaster will verify the first obituary match by making a call or visit to the family. If it is determined from the family that John Smith did not take Penicillin and did not die from respiratory arrest, the broadcaster would proceed to the family of Alan Black, then Hugh Tremblay, and Joe White. The ability to verify matches (for example, the willingness of families to provide personal information to the broadcaster), and the resources that the broadcaster has available to verify matches (for example, the broadcaster may have resources to only verify two matches and therefore if a correct match is not found after two attempts, then they would give up and that would be considered a no match), affects the re-identification probability.

The broadcaster may not be able to contact a family, in which case it is not possible to verify a match. The family may respond but not be truthful with the broadcaster if they do not want their experiences to be publicized or perceive the broadcaster’s questions to be an invasion of their privacy. Therefore, the ability to verify a match is probabilistic.

It is important to note that the re-identification probability here is semantically different from the probability of a random match for an unmotivated adversary. In the case of a random match, the unmotivated adversary cannot be certain which of the matches is correct, but can associate a probability to each match. For the motivated case, it depicts the probability of having a certain match, in other words, it is the probability of the adversary being able to verify the correctness of one of the potential matches.

There are currently no re-identification risk measures that take into account the process of multiple matching attempts with verification. However, some adversaries in the real world incorporate verification and multiple attempts in their re-identification process, making it critical to mimic this actual behaviour in measures of re-identification risk.

### Notation

The adversary will match against obituaries. This is relatively easy because there are obituary aggregators and meta search engines [31-33]. We make an assumption that there exists a registry of all deaths that is used for matching by the adversary (the implications of this assumption are discussed in the Limitations section).

The individuals in this obituary are members of the set Z and the reports in ADE are members of the set *U*. The adversary matches on the set of quasi-identifiers that are common to the ADE database and the obituary. The discrete variable formed by cross-classifying all values on the quasi-identifiers is *x*. Each one of these values is an equivalence class. The set of equivalence classes is denoted by *J*. Let *y*_*j*_ denote the value of an equivalence class in *J*, such that *y*_*j*_ ∊ *J*. Let *x*_*Z,i*_ denote the value of *x* for individual *i* in set Z. For example, if we have two quasi-identifiers, age and gender, then we may have an individual characterized as *x*_*Z*,1_ = [50, "*MALE*"]. In this case fifty year old males would be one of the equivalence classes.

The frequencies for different values of the quasi-identifiers are given by $F_{j}=\sum_{i=1}^{\left|Z\right|}I\;\left(x_{Z,i}=y_{j}\right)$, where I (·) is the indicator function, |Z| represents the size of the set Z, and *F*_*j*_ is the size of an equivalence class in the set Z. Similarly we have $f_{j}=\sum_{i=1}^{\left|U\right|}I\;\left(x_{U,i}=y_{j}\right)$, where ƒ_*j*_ is the size of an equivalence class in the set *U*. Some values of ƒ_*j*_ are zero because not all equivalence classes in the obituaries will have reported deaths in ADE. In what follows we assume that ƒ_*j*_ ≤ *F*_*j*_, and that information is recorded consistently in both files (i.e., there are no errors or duplicates). Moreover, to simplify our analysis, we assume that no two records in within each of *U* or *Z* are identical.

**Figure 1:**
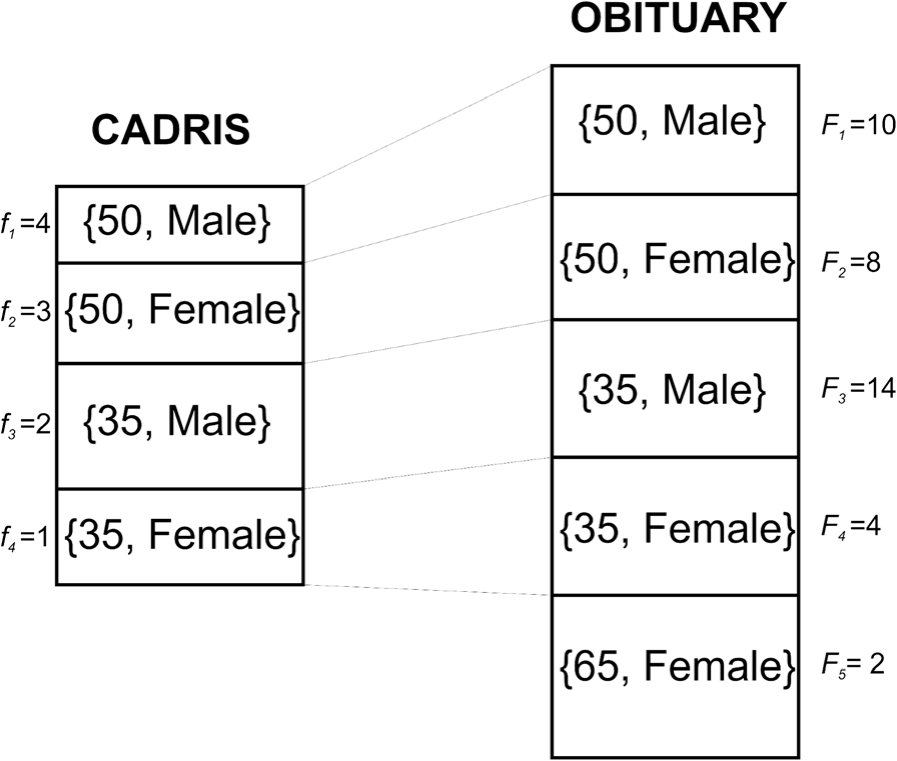
**Example to illustrate how the percentage of ADE records at risk are computed from the matched equivalence classes.** In this example we assume there are only two quasi-identifying attributes: age at death and gender.

For example, consider the hypothetical datasets in Figure 1. The set *U* is the ADE database, and this has four equivalence classes. The first equivalence class of 50 year old males has a size of 4 (i.e., ƒ_1_ = 4). The fourth equivalence class of 35 year old females has only a single record (i.e., ƒ_4_ = 1). The Z set is the obituary and that has 5 equivalence classes. The first equivalence class of 50 year old males has ten records (i.e., F_1_ = 10).

### Measurement of re-identification risk

In this paper we formulate the re-identification risk for the motivated adversary who requires matches to be verified. Re-identification risk is defined as the probability of a correct match, given the number of verification attempts the adversary is willing to do. This number depends on how motivated the adversary is: a mildly motivated adversary might stop after one verification attempt, while a highly motivated adversary might persist and verify as many potential matches as possible, limited only by financial or practical considerations. The formulation of the risk is a function of the number of verification attempts the adversary is willing to try.

We denote by *P* the probability of being able to verify whether a matching attempt is correct or incorrect. For example, if the value of *P* is 1, then the adversary is always able to verify if a match was correct or not. If the value of *P* is 0, then the adversary is not able to verify any match. The adversary will select a single individual of interest from the ADE database with value *x*_*U,i*_ (most likely *x*_*U,i*_ is chosen to correspond to the smallest equivalence class size in *Z*) and will attempt to match this record with the individuals in *Z*. The record selected might have certain characteristics that are of particular importance to the adversary (such as died while taking a particular medicine). Denote by *F*_*j*_ the size of the equivalence class in *Z* with the same quasi-identifier values as *x*_*U,i*_. We assume that the adversary has no prior information about any of the individuals in *U*. We assume that the adversary will proceed sequentially through the potential matches in *F*_*j*_ until the correct match is found (the order of the records does not matter). If a match is found, then we say that the adversary stops the verification process. Moreover, we assume that the adversary will attempt verification for only one individual. In other words the adversary will stop the verification process if: (1) a verification is achieved, or (2) if all possible matches are attempted (*F*_*j*_ attempts) with no success.

Denote by $P_{j}^{n}$ the probability that the correct match was discovered after performing exactly *n* verifications (note that *n* ≤ *F*_*j*_). Then, we have:

#### Lemma 1

The probability of finding the correct match at the *n*^th^ attempt, $P_{j}^{n}$, is given by:

(1) $$\begin{array}{l} P_{j}^{n}=\left\{\begin{array}{cc} \;\frac{p}{F_{j}} & ,\mathrm{if}\;F_{j}>n+1\\ \begin{array}{l} \;\frac{p}{F_{j}}+\frac{p^{F_{j}-1}}{F_{j}}\\ \;\frac{\left(1-p^{F_{j}-1}\right)p+p^{F_{j}-1}\left(F_{j}-1\right)\left(1-p\right)}{F_{j}} \end{array} & \begin{array}{l} ,\mathrm{if}\;F_{j}=n+1\\ ,\mathrm{if}\;F_{j}=n\; \end{array} \end{array}\right) \end{array}$$

Note that, particular attention was given for the cases where *F*_*j*_*∊* {*n*-1,*n*} because:

1. if the adversary performs *F*_*j*_ –1 attempts that all result in verified non-matches, then there is no need for any further verifications as this implies that the last record will be a match.

2. if the adversary performs *F*_*j*_ attempts, and if *F*_*j*_ –1 of these result in verified non-matches and only one attempt was unverifiable, then we can deduce the sole unverified record is the correct match.

The proof is included in Additional file 1.

Now, if we assume that *M* is the maximum number of attempts that the adversary is willing to try, and if *M*_*j*_ = min(*M,F*_*j*_). In other words, *M*_*j*_ is the maximum number of attempts that the adversary can perform for class *F*_*j*_, either because of his own limitations, i.e., when *M* ≤ *F*_*j*_, or because of the equivalence class size limitation, i.e. when *F*_*j*_ *< M*. Then we have:

#### Lemma 2

The risk of the adversary getting a successful match in *M*_*j*_ attempts and knowing (through verification) that s/he has the correct match is:

(2) $$R_{j}=\left\{\begin{array}{cc} M_{j}\frac{p}{F_{j}} & \;,\mathrm{if}\;F_{j}>M_{j}+1\\ \begin{array}{l} \;M_{j}\frac{p}{F_{j}}+\frac{p^{F_{j}-2}}{F_{j}}\\ \;p+p^{F_{j}-1}\left(1-p\right)\\ \;1 \end{array} & \begin{array}{l} \;,\mathrm{if}\;F_{j}=M_{j}+1\\ \;,\mathrm{if}\;F_{j}=M_{j}\;\\ \;,\mathrm{if}\;F_{j}=1 \end{array} \end{array}\right)$$

The proof is included in Additional file 1.

**Figure 2:**
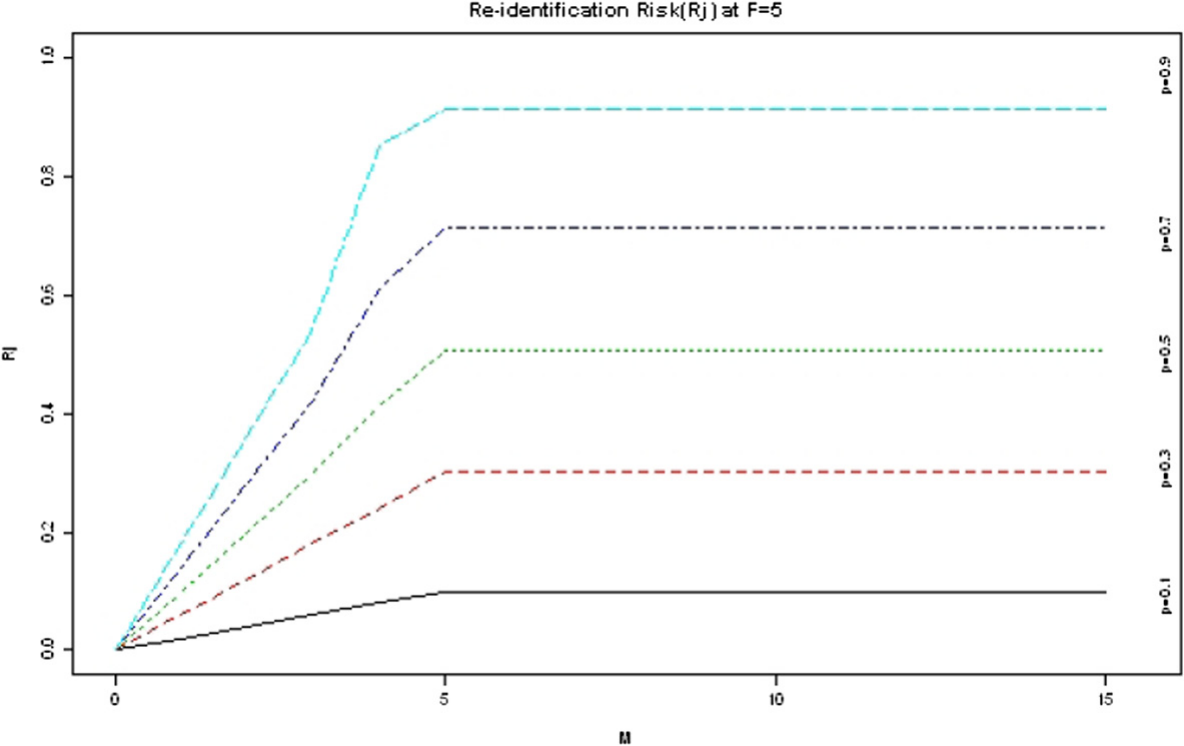
**Plot showing how the re-identification risk, *R***_***j***_**, varies with the number of attempts (*M*) and the probability of being able to verify a match (*p*).**

Figure 2 shows the risk *R*_*j*_ as a function of *M* and *P* with *F*_*j*_*=5*. Note that for a given *P*, the risk increases with the increase in *M* until *M* = *F*_*j*_*=5*, at that point, *R*_*j*_ becomes insensitive to the increase in *M*. Note also that *R*_*j*_ is very sensitive to the change in the *P* value, for example, for *M* = 4, *R*_*j*_ jumps from 0.08 at *p* = 0.1 to 0.85 at *p* = 0.9.

If an adversary has substantial resources and will not stop matching and verifying until s/he goes through all of the matching records in Z, then we can assume that *M*_*j*_ = *F*_*j*_. In our example, the broadcaster was willing to contact all of the matching records in the obituaries to determine which was the correct family. In such a case the re-identification risk is equal to *p* + *p*^4^ (1–*p*), meaning that the risk depends mainly on the ability to verify the matches. In fact, with p = 0.9 and *M*_*j*_ = *F*_*j*_, the risk is 0.965. If the broadcaster has or can afford good staff who are quite successful at verifying matches, then the re-identification risk can be quite high.

### Applications of the risk model

The data custodian can use the above re-identification risk model to evaluate the risk of re-identification. However, in applying the model in practice, the custodian must answer a number of questions. We will address these questions below.

#### What type of adversary to assume?

In practice, the data custodian will not know a priori which type of adversary is most likely: will it be an adversary who is content with an unverified match (assuming no uniques in the obituaries) or an adversary who needs to have verified matches. If it is an adversary who will not verify matches, the probability of re-identification as presented in [34] is: Rj′=1Fj. If the adversary needs to verify matches, then the probability of re-identification is given in Lemma 2: *R*_*j*_.

#### How can an adversary verify a match?

An adversary can contact neighbourhood businesses and individuals, and directly call potential matches to verify if a match was correct. In the case of the press, members of the public are often willing to reveal information to reporters in the hope or expectation of being part of the story. If individuals are not cooperative, many social engineering techniques exist [35-37], and have been used to obtain very personal information from individuals and organizations (as well as to commit more dramatic crimes such as bank robberies) [38,39]. A recent review of data breaches indicated that 12% of data breach incidents involved deceit and social engineering techniques [40]. It has been argued that the main threat to medical privacy is social engineering, and such techniques have been used by private investigators to surreptitiously obtain health information [41,42]. For example, one can use social engineering techniques to verify identity by pretending to be from a bank checking on an unusual transaction, impersonate someone collecting on a medical bill, pretend to be someone from the health insurer verifying some details otherwise an insurance policy will expire or a claim will not be paid, or act as a receptionist from a hospital/clinic confirming an appointment.

#### How many verification attempts will there be (M)?

In this paper, we analyse the case of a motivated adversary who will attempt to verify matches. The number of verification attempts depends on the level of motivation. A mildly motivated adversary might only attempt one verification: *M*_*j*_ = 1. While a more motivated adversary might take further measures to verify potential matches. A highly motivated adversary might attempt to verify all potential matches: *M*_*j*_ = *F*_*j*_.

##### Who is a motivated adversary?

One way to determine the number of verification attempts by an adversary, *M*, is to examine the financial value of the re-identification to the adversary and assume that the adversary would not consume more resources on the re-identification than the information is worth. In the underground economy, the rate for the basic demographics of a Canadian has been estimated to be $50 [43]. Another study determined that full-identities are worth $1-$15 [44]. However, an adversary will already have access to a public registry with the identity information to match with. For example, in the case of the ADE database, the obituaries would contain the identity information for the individuals, and there is evidence that such information is sufficient to create new identities for the deceased individuals and resell them [45]. The matching with the ADE database does not add new information. Consequently, we cannot use the value of identity data as a driver for deciding on *M*.

The re-identification of patient records exposes patients’ health information. There is evidence that a market for individual medical records exists [46,47]. This kind of identifiable health information can also be monetized through extortion, as demonstrated recently with hackers requesting large ransoms [48,49]. In one case, where the ransom amount is known, the value per patient’s health information is $1.20 [49]. However, given that minimum wage is $8.75 per hour in Ontario, at such a low patient record value an adversary would not be financially incented to spend more than a few minutes trying to verify a match. Consequently we can assume that where the driver is the financial value of the patient record that *M*_*j*_ = 1.

If the motivation is not financial then the adversary may expend more effort and hence increase *M*. For example, for a public data set the adversary may be in the media and doing an investigation, as in our example of the broadcaster. The adversary may be performing a demonstration attack to show that records in a dataset can be re-identified [3]. In those cases the individuals who are being contacted may not appreciate the intrusive questions or someone may suspect a social engineering scheme, which means that there is a deterrent from having an *M* that is too large. If we are to use known real attack examples where verification was performed, then an *M* in the range of 10 or 15 would seem reasonable.

## Methods

Given the number of verification attempts, *M*, an adversary is willing to make, the probability of verification *p*, and an acceptable risk threshold *τ*, we provide recommendations (in the Discussion Section) to the data custodian on the minimal population class size needed to have an acceptable risk.

But before that, in this and the following section, we conduct a simulation to estimate the proportion of individuals in the ADE dataset (having an outcome of death) with a high probablity of re-identification by linking to obituaries. This estimate was performed for different combinations and levels of granularity of quasi-identifiers. For the simulation, we focused on the two extreme cases of a mildly motivated adversary, and a highly motivated one.

### Datasets

We performed a simulation using mortality data from Statistics Canada for the years 1997 to 2005 [50,51]. Under the assumption that there exists a comprehensive obituary registry that is available to an adversary, we computed the re-identification risk for Canadian ADE reports with death outcomes over this period. We used the Statistics Canada mortality data to simulate such a comprehensive obituary dataset. We refer to this as the simulated obituary.

In total there were 1,993,351 deaths during the study period. Statistics Canada provides two death files that are not linked. The first reports the age at death, gender, province, and year of death. The second file reports the province, month, and year of death. We created 1000 simulated obituaries by distributing the deaths in the first file by month with the same proportions as in the second file. We then distributed the deaths to the days within each month using a uniform distribution. All analysis results were averaged across the 1000 simulated obituaries.

The relevant outcomes in the ADE dataset were deaths, whether they were related or not to the drug. During that same period there were 3,482 deaths reported in the Canadian ADE database.

**Figure 3:**
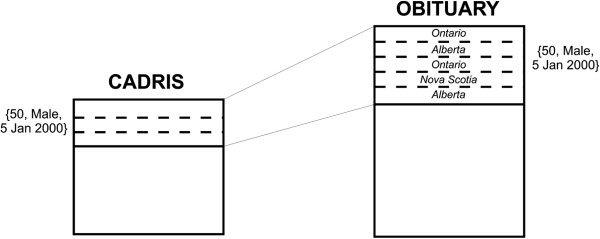
An example illustrating how province is assigned to the ADE dataset from the virtual obituary file.

The ADE dataset made available by Health Canada does not include the province. Therefore, for every equivalence class in a simulated obituary, we assigned the province randomly to ADE records. This is illustrated in Figure 3 for 50 year old males who died on 5^th^ January 2000. The obituary has five records with two deaths in Ontario, two in Alberta, and one in Nova Scotia, and the ADE database has three records of unknown province. We therefore assigned the first ADE record the province according to the distribution in the obituary equivalence class (i.e., Ontario with a probability of 0.4, Alberta with a probability of 0.4, and Nova Scotia with a probability of 0.2). This was repeated for the next ADE record with an adjusted probability depending on the assignment for the first ADE record, and so on.

The ADE dataset has the date of reporting. This may not be the exact date of death. Manufacturers and distributors are required to report deaths within fifteen days for marketed products. Therefore, when matching the ADE dataset with the simulated obituaries, we consider deaths within fifteen days of the reporting date to be in the same equivalence class.

### Ethics

The datasets used in this analysis were public data sets available from Health Canada and Statistics Canada. According to guidance received from our research ethics board, no ethics review is required for studies utilizing public data sets.

### Risk measurement

For each equivalence class in the simulated obituary we computed the re-identification risk using Equation (2). If the risk was higher than a threshold *τ* then we considered all of the individuals in that equivalence class in the ADE dataset to be at an elevated risk of re-identification by matching the ADE report with the obituary. Consider the hypothetical example in Figure 1. Here the ADE database only has 4 equivalence classes that are matched to four equivalence classes in the simulated obituary. The fifth equivalence class in the simulated obituary (65 year old females) is not considered at all in our analysis because there are no such equivalence classes in the ADE database. If we find that *R*_1_ > τ and *R*_3_ > τ, then the percentage of ADE records at risk would be computed as 100×f1+f3f1+f2+f3+f4. This value would then be averaged across the 1000 simulated obituaries.

### Risk threshold

What should the value of *τ* be? Though a minimum equivalence class size of 3 is often suggested [52-55], a common disclosure control recommendation in practice (ie., in data release policy and guidance documents) is to ensure that equivalence classes have at least 5 records [20-26,56-58]. This translates to a practical *τ* = 0.2, which we will use in our analysis.

We used different values for *p* to reflect potentially different challenges in verifying a match. These were 0.5, 0.7, and 0.9. We did not use values below 0.5 because we know from the ADE dataset and the national broadcaster example that verification is doable relatively easily, which makes it difficult to justify a lower value for *p*.

For the number of attempts, *M*_*j*_, we use *M*_*j*_ =1 for a mildly motivated adversary, and *M*_*j*_ = *F*_*j*_ for an adversary who will exhaust all matching records in the obituary. By examining Equation (2), we see that *R*_*j*_ > *p* when *M*_*j*_ = *F*_*j*_, and therefore the inequality *R*_*j*_ > *τ* holds true. In fact, unless *p* < 0.2 an adversary that tries to verify all potential matches will always result in a high risk of re-identification for all records in the ADE database. Therefore, there is no need to empirically test the case where *M*_*j*_ = *F*_*j*_.

#### Quasi-identifiers

The percentage of ADE records at risk was computed for different combinations of quasi-identifiers at different levels of precision. The original quasi-identifiers included province, age at death in years, gender, and date of reporting. Age at death was generalized to five year intervals and ten year intervals. The date of reporting was generalized to month and year, and only year. This gave us a total of twelve quasi-identifier combinations.

For our simulation, we looked at these different combinations of quasi-identifiers and how they affected the percentage of records at risk. Note that the granularity of the quasi-identifiers affects the size of the population equivalence classes and the re-identification risk as a result.

### Interpretation

In the Discussion, we calculate the minimal equivalence class size required in the obituaries that guarantees 0% of records at risk. However, it is important to note that previous disclosures of cancer registry data have deemed thresholds of 5% and 20% registry members at risk as acceptable for public release and research use respectively [26,27].

## Results

The percentage of ADE deaths (averaged across the 1000 simulated obituaries) are given in Table 4. These results assume an adversary will attempt only one verification.

**Table 4:** The percentage of ADE deaths that are at a high risk of re-identification by matching to an obituary for different combinations of quasi-identifiers

**Quasi-identifiers**						**ADE records at risk (%)**		
**Province**	**Age at death**	**Gender**	**Day of report**	**Month of report**	**Year of report**	**p = 0.5**	**p = 0.7**	**p = 0.9**
	X	X	X	X	X	1.95	3.46	5.05
	X	X		X	X	0	0	0
	X	X			X	0	0	0
	2 yr	X			X	0	0	0
	5 yr	X			X	0	0	0
	10 yr	X			X	0	0	0
X	X	X	X	X	X	18.44	25.17	30.78
X	X	X		X	X	0.21	0.4	0.63
X	X	X			X	0.12	0.24	0.39
X	2 yr	X			X	0.04	0.07	0.13
X	5 yr	X			X	0.02	0.03	0.05
X	10 yr	X			X	0	0	0.01

We considered age converted into a 2 year interval, 5 year interval, and a 10 year interval.

By including all of the quasi-identifiers, including the province, the percentage of ADE deaths at risk varies from 18.44% to 30.78% depending on the assumption about verification probability. Therefore, under the most lenient assumption of *p* = 0.5, the risk of re-identification would still be considered high. The removal of province does reduce the percentage of ADE deaths at risk to a range from 1.95% to 5.05%. This is clearly a significant reduction in risk and is quite close to the 5% limit deemed acceptable for public disclosure.

The removal of the exact day of reporting (and having only month and year) ensures that the percentage of ADE records at risk is from 0.21% to 0.63%, which is very low, even if the province field is included. In fact, that would seem to be the most sensible approach to disclosing the ADE data and ensuring acceptable risk. This allows the retention of the province field as well as age at death in years.

## Discussion

### Summary

We have constructed a re-identification risk model that mimics the behaviour of an adversary who is motivated to varying degrees to verify potential matches, and captures the realistic situation where the probability of verification of a match is not always one. We then applied this model to evaluate the risk of re-identification of post-market adverse drug event data in Canada. Our analysis focused only on cases where the outcome being reported is death because for other events the plausibility of a re-identification is very limited.

Under the first scenario we considered, the adversary is mildly motivated to the extent that they would be prepared to make *M* = 1 attempts at verification. In this case, the inclusion of the province and the full reporting date will result in a large percentage of individuals at a high risk of re-identification. However, the removal of the exact reporting day, and disclosing only the month and year of the ADE report ensures that the risk is always low.

For the second scenario where the adversary is highly motivated and will attempt to verify all matches, we showed that the risk of re-identification per record will always be higher than the common 0.2 threshold (no matter how generalized the quasi-identifiers are). The probability of being able to verify a match, must be smaller than 0.2 for the risk of re-identification to be lower than the threshold.

In Table 5 we show the minimum equivalence class size. Under the second scenario an adversary would have to verify at most all of these matches. If we assume that the date of reporting will not be disclosed but that province will be, then this means that more than 6,000 matches would have to be verified. Arguably, even if the adversary is highly motivated, verifying that many matches is not realistic, as it would be quite costly and hence would act as a strong deterrent for attempting to verify all matches.

**Table 5:** The minimum size of an equivalence class in a simulated obituary

**Quasi-identifiers**						**Minimum obituary**
**Province**	**Age at death**	**Gender**	**Day of report**	**Month of report**	**Year of report**	**Equivalence class size**
	X	X	X	X	X	263
	X	X		X	X	6514
	X	X			X	8459
	2 yr	X			X	16671
	5 yr	X			X	39780
	10 yr	X			X	79619
X	X	X	X	X	X	253
X	X	X		X	X	6486
X	X	X			X	8459
X	2 yr	X			X	16671
X	5 yr	X			X	39780
X	10 yr	X			X	79619

In what follows, we provide recommendations on the quasi-identifiers level of granularity that will ensure low re-identification risk for both the highly motivated and mildly motivated adversaries (and hence all the motivation levels between the two). Then we provide more specific recommendations on the minimal population class size required for a given number of verification attempts, *M*, a given probability of verification *p*, and an acceptable risk threshold *τ*.

#### Practical recommendations

Our primary recommendation for the ADE database is that the province field can be disclosed, but not the exact date of reporting. This will ensure that the overall risk of re-identification is quite low under the scenario of a mildly motivated adversary. For a highly motivated adversary, the equivalence classes after implementing the above recommendation are sufficiently high to act as a practical deterrent to attempting to verify all matches. In other words, setting *M*_*j*_ = *F*_*j*_ is not realistic in these cases.

While our analysis was specific to the Canadian ADE database, more general recommendations can be provided. Specifically, policies to help reduce the probability of a successful verification are necessary. These will help reduce the value of *p* and either act as a deterrent for adversaries to attempt verification, or reduce the chances of success if they do attempt verification.

First, to the extent possible, the public should be discouraged from divulging personal information so readily. Also commercial and government organizations should be discouraged from collecting non-required personal information, for doing so makes it more acceptable for the public to divulge personal information to complete daily transactions. Healthcare organization staff should be educated about social engineering techniques that an adversary can use to gather information about patients or employees, and should be trained to check requests for information to ensure that they are legitimate.

Second, data custodians can generalize their data to increase the costs of verification for an adversary. This can create a financial deterrent. A description of how to control the dataset equivalence class sizes through generalization of the quasi-identifiers is described in the next section.

### Controlling re-identification risk

Re-identification risk can be controlled for the adversaries with all levels of motivation. This can be achieved by computing the smallest value of *k* that all *F*_*j*_ can assume. Once such a value is set, techniques described in [34] can be used to de-identify the data set, such as the ADE database, before its public disclosure.

Given the risk threshold *τ*, and given values for *M* and *p*, the data custodian would want to manage the risk of re-identification before disclosing the ADE or other dataset without having to perform an extensive simulation looking at all possible generalizations of quasi-identifiers. The theorem below provides the values of *k* that ensures that no record in the disclosed dataset is at risk of re-identification higher than the threshold.

#### Theorem 1

For given values for *M* and *p*, the smallest value *k* that *F*_*j*_ can assume is:

$$k=\max\left(M+2,\left\lceil \frac{\text{Mp}}{\tau }\right\rceil +1\right)$$

The proof is provided in the Additional file 1.

**Figure 4:**
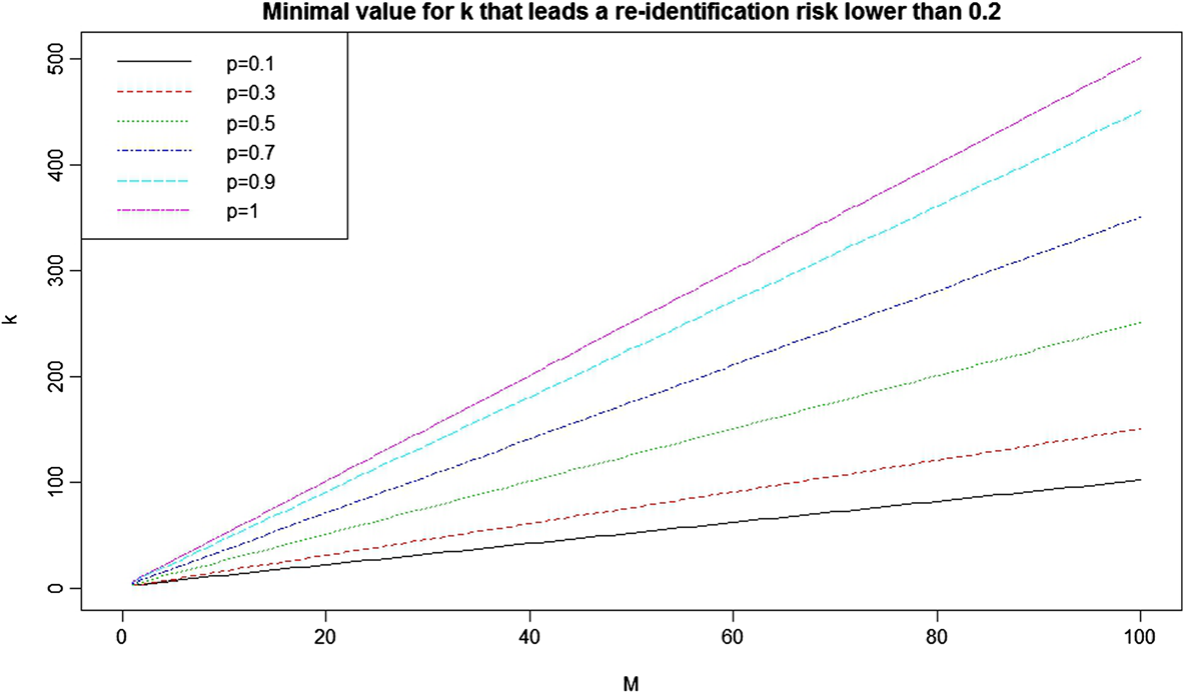
The relationship between M, p, and k.

The relationship between *M*, *p*, and *k* is illustrated in Figure 4. There is a linear relationship between *M* and *k* whose steepness varies by *p*. Note that, for the persistent adversary (high *M* value), *k* would be set sufficiently high that it would act as a deterrent from attempting to verify all matches.

Under the extreme circumstance, if we have a highly motivated adversary (*M* has no upper limit) and if *p* = 1, then even if we generalize all records in the dataset to one class (or even if we don't release anything), the adversary will call the whole population and know who has what since *p* = 1.

### Related work

Previous work has assumed that an adversary would have prior knowledge which she uses to re-identify a single record. There is also work considering prior knowledge by the adversary about multiple records in the dataset. For example, prior knowledge can be modeled as knowledge of the sensitive values in a dataset that can then be used to eliminate candidate matches [59]. This is referred to as “corruption”. Say if there are four individuals who are 44 year old and male, but the adversary already knows that two of them died from a car accident rather than a death related to taking a drug, then they can be eliminated from the matching a priori. The concept of corruption models a different type of attack whereby the adversary already has prior sensitive information about some of the records in an equivalence class. Whereas in our context the adversary only gains this sensitive information through a process of verification: the adversary does not have prior knowledge about any of the individuals in the disclosed database.

In other work prior knowledge can be modeled as a belief function over the possible values of the quasi-identifiers [60,61]. Here again this quite detailed knowledge is available to the adversary prior to the attack.

In our case we assume that the adversary has minimal prior knowledge as this is similar to the context of the re-identification attack on the ADE database. The broadcaster did not have prior information about the drugs that certain individuals in the ADE database have taken or their reactions to these drugs, and did not utilize prior belief functions on the variables in the ADE database. The broadcaster did not know the identity of any of the individuals in the ADE database prior to launching an attack.

Therefore, this prior work is modeling a different method of attack than the one that has actually occurred in this case. There is no evidence of real-world re-identification attacks that have used such extensive prior information.

### Assumptions and limitations

There are two general approaches to generalization (or “recoding”): global and local [62-67]. With global recoding all the values for a particular variable are generalized the same way. For example, a date of birth is generalized to year of birth for all records. With local recoding the generalization levels can differ among records. For example, some records can have a month and year generalization for the date of birth while others can have a five year range. Our model assumes global recoding, which is consistent with the most common forms of generalization that are used in practice. De-identified data sets with local recoding are often more difficult to analyze because standard statistical methods cannot be applied to them. Also, note that in the release of the ADE database and in discussions about its de-identification in the relevant court documents, all generalization discussions and examples pertained to global recoding.

One limitation in our analysis is that we had to create simulated obituaries. In doing so, we assumed that deaths occur uniformly within any month of the year, although we did account for variation in the distribution of deaths across months.

We also assumed that comprehensive obituaries exist. This assumption means that we have obtained results that inflate the risk measures. To the extent that real obituaries are incomplete, the risk of re-identification would be lower than reported here. Therefore, our risk results should be considered higher than they would be in the non-ideal real world.

The choice of *p* is a challenge in practice. However, given the precedents and the ease of using social engineering techniques to extract information from individuals, it would be prudent to set this value relatively high. It is also relatively easy to perform a sensitivity analysis on the choice of *p* to determine whether plausible values would affect conclusions drawn about the risk of disclosing the data.

In our model we set *p* as a constant for all equivalence classes. This is not necessarily true because certain equivalence classes may be easier to verify. However, there is no evidence or data to indicate which equivalence classes would be easier, and thus is a hypothetical rather than a real concern.

Another limitation is that our study focused on re-identification risk from ADE reports in Canada and these empirical results may not generalize to other jurisdictions. In particular, the FDA is setting up a sentinel network to collect data from 100 million patients to provide more immediate, complete, and detailed information about ADEs [68,69]. To the extent that this data will be accessible broadly, re-identification risk assessments should be considered because this represents information on one third of the US population, which is a much larger sampling fraction than for the Canadian ADE database.

## Conclusions

In this paper we developed a new model to measure re-identification risk that takes into account the adversary’s ability to verify matching. This is arguably a more realistic model of how re-identification attacks are actually performed. We then applied this model to the re-identification of Canada’s adverse drug event database. This analysis provided evidence-based guidance on what information can be publicly disclosed while maintaining the privacy of the data subjects.

## Endnote

^a^ There are exceptions where the disclosure is mandated by law, e.g., for some communicable diseases, or otherwise permitted under the discretion of the data custodian.

## Competing interest

KEE performs consulting work for healthcare providers and governments on best practices for the de-identification of health data, and has a financial interest in Privacy Analytics Inc., which develops de-identification software. The remaining authors do not have any conflicts to declare.

## Authors’ contributions

KEE designed the study, performed data analysis, and wrote the paper. FD designed the study, performed data analysis, and wrote the paper. AN performed data analysis. EJ performed the literature review and participated in writing the paper. All authors approved the final version of the paper.

## Pre-publication history

The pre-publication history for this paper can be accessed here:

http://www.biomedcentral.com/1472-6947/13/114/prepub

## Supplementary Material

Additional file 1 Contains the proofs for the lemmas and theorems in the main text of the article. Click here for file
