# Supplementary material for: Evaluating the risk of patient re-identification from adverse drug event reports
Source: BMC Med Inform Decis Mak. 2013 Oct 5;13:114. doi: 10.1186/1472-6947-13-114 (PMC4137558; doi:10.1186/1472-6947-13-114)
Supplement: Additional file 1 — Contains the proofs for the lemmas and theorems in the main text of the article. [file 1472-6947-13-114-S1.pdf]

Appendix A: Proofs

This appendix contains the proofs for the lemmas and theorems in the main text of the article.

A.1 Proof of Lemma 1

Denote by:

1. A_h, the fact that the hth record is the match, and by $\neg A_h$ the fact that the hth record is not a correct match
2. B_h, the fact that the hth record can be verified (as a match or non-match), and by $\neg B_h$ the fact that the hth record can not be verified.

Note that, the adversary reaches the nth attempt only when every previous attempt was either unverifiable or verified as a non-match.

Consider first the case where $F_j > n+1$, the probability of getting a successful match from the ***n*^th^ attempt,** P_j^n , is the probability that the nth record is the match and that the match can be verified and that the previous records attempted were either verified as non-match or unverified

$$P_j^n = \Pr\{A_n \wedge B_n \wedge (\bigcap_{h=1}^{n-1}[(\neg A_h \wedge B_h) \vee (\neg B_h)])\}$$

However, because there is only one correct match, we get:

$$P_j^n = \Pr\{A_n \wedge B_n \wedge (\bigcap_{h=1}^{n-1}[(\neg A_h \wedge B_h) \vee (\neg A_h \neg B_h)])\}$$

$$= \Pr\{A_n \wedge B_n \wedge (\bigcap_{h=1}^{n-1}[B_h \vee \neg B_h])\}$$

$$= \Pr\{A_n \wedge B_n\}$$

$$= \frac{p}{F_j}$$

When $F_j - 1 = n$, then we need to consider two cases:

1. The case where the *n*^th^ attempt is the match, and
2. the case where all the $F_j - 1$ attempts result in verified non-matches, as this implies that the last record will be the match (without having to go any further than *n* attempts).

$$P_j^n = \Pr\{A_n \wedge B_n \wedge (\bigcap_{h=1}^{n-1}[(\neg A_h \wedge B_h) \vee (\neg B_h)])\} + \Pr\{\bigcap_{h=1}^{n}(\neg A_h \wedge B_h)\}$$

$$= \Pr\{A_n \wedge B_n\} + \Pr\{A_{n+1} \wedge \bigcap_{h=1}^{n} B_h\}$$

$$= \frac{p}{F_j} + \frac{p^{F_j - 1}}{F_j}$$

When $F_j = n$, then we need to consider two cases

1. the case where, out of the F_j attempts performed, $F_j - 1$ of these result in verified non-matches and only one attempt was unverifiable, in such case, we can deduce the sole unverified record is the correct match.
2. The case where the nth attempt is the match that was not discovered in the previous attempt (i.e. we need to discard the case where the $F_j - 1$ previous attempts resulted in verified non-matches)

$$P_j^n = \Pr[A_n \wedge B_n \wedge \exists i \in \{1,..,n\} s.t. \neg B_i] + \Pr[(\neg A_n \wedge B_n) \wedge (\bigcup_{i=1}^{n-1}(A_i \wedge \neg B_i \wedge B_h \text{ for some } h \neq i)]$$

Where $\exists i \in \{1,..,n\} s.t. \neg B_i$ means that at least one of the previous matches was unverifiable.

$$P_j^n = (1 - p^{F_j - 1})\frac{p}{F_j} + \frac{(F_j - 1)p(1-p)p^{F_j - 2}}{F_j}$$

A.2 Proof of Lemma 2

We consider each of the different cases separately:

1. if $F_j > M_j + 1$, then

$R_2^j = P_j^1 + P_j^2 + ... + P_j^{M_j}$ From Lemma 1 we get:

$$R_2^j = M_j \frac{p}{F_j}$$

2. if $F_j = M_j + 1$, then:

$$R_2^j = P_j^1 + P_j^2 + \ldots + P_j^{M_j - 1} + P_j^{M_j}$$

Hence, from Lemma 1, we get:

$$R_2^j = M_j \frac{p}{F_j} + \frac{p^{F_j - 2}}{F_j}$$

3. if $F_j = M_j$ then:

$R_2^j = P_j^1 + P_j^2 + \ldots + P_j^{M_j - 2} + P_j^{M_j - 1} + P_j^{M_j}$ then from Lemma 1 we get:

$$R_2^j = (M_j - p^{F_j - 1}) \frac{p}{F_j} + \frac{p^{F_j - 1} + p^{F_j - 1}(F_j - 1)(1-p)}{F_j} = p + p^{F_j - 1}(1-p)$$

A.3 Proof of Theorem 1

Recall that, the risk is the following:

$$R_2 = \begin{cases} M'\frac{p}{F'} & , if\ F' > M'+1 \\ M'\frac{p}{F'} + \frac{p^{F'-2}}{F'} & , if\ F' = M'+1 \\ p + p^{F'-1}(1-p) & , if\ F' = M' \\ 1 & , if\ F' = 1 \end{cases}$$

Where $F' = \min_j F_j$ and $M' = \min\left(M, \min_j\left(F_j\right)\right)$.

Given the values for M and p, we need to find the smallest value for F_j that satisfies $R_2 \leq \tau$.

Observe that, if $F_j \geq M + 2$ for all j, then it is enough to have $M\frac{p}{F_j} < \tau$ for all j.

Hence we can set our k to be

$$k = \max\left(M + 2, \left\lceil \frac{Mp}{\tau} + 1 \right\rceil\right)$$
